# Transcriptomic Analysis of Rice (Jijing129) Reveals Growth and Gene Expression Responses to Different Red-Blue Laser Light Treatments

**DOI:** 10.3390/plants14243712

**Published:** 2025-12-05

**Authors:** Xuemei Liang, Qi Liu, Li Qin, Peng Jia, Jianfeng Wang, Changjiang Zhang, Xintong Dai, Wenbo Yu, Xiaoyu Lei, Ningning Wang, Minglai Yang

**Affiliations:** 1 College of Information and Technology, Jilin Agricultural University, Changchun 130118, China; xuemeil@jlau.edu.cn (X.L.); 20231617@mails.jlau.edu.cn (Q.L.); daixintong20@163.com (X.D.); 20221137@mails.jlau.edu.cn (W.Y.); 20231258@mails.jlau.edu.cn (X.L.); 2 State Key Laboratory of Luminescence and Application, Changchun Institute of Optics, Fine Mechanics and Physics, Chinese Academy of Sciences, Changchun 130033, China; qinl@ciomp.ac.cn (L.Q.); jiapeng@ciomp.ac.cn (P.J.); 3 Key Laboratory of Facility Vegetable of Jilin Province, Jilin Academy of Vegetable and Flower Sciences, Changchun 130119, China; hortwjf@163.com; 4 Faculty of Agronomy, Jilin Agricultural University, Changchun 130117, China; 17861922969@163.com

**Keywords:** RNA-seq, light quality, red: blue ratio, differentially expressed genes (DEGs), nitrogen metabolism, photosynthesis

## Abstract

Artificial supplemental lighting represents a crucial agricultural technique for enhancing plant growth and development, with researchers continuously investigating the effectiveness of various light sources in horticultural applications. Laser technology, characterized by its monochromatic nature, high coherence, and elevated energy density, presents a promising light source whose potential applications and underlying mechanisms in plant supplemental lighting remain to be thoroughly explored. To investigate the effects of different red-to-blue light ratios in laser supplemental lighting on rice (*Oryza sativa* L. cv. Jijing129) seedlings, we conducted a seedling-stage lighting experiment on the rice cultivar Jijing129 in a greenhouse using an LGI-660/450 dual-wavelength semiconductor laser system. The experimental design included a natural light control (AL) and three laser treatment groups, with red: blue (R:B) ratios and corresponding photon flux densities as follows: BL (50:50; 150:150 μmol m^−2^ s^−1^), CL (60:40; 180:120 μmol m^−2^ s^−1^), and DL (75:25; 225:75 μmol m^−2^ s^−1^). We systematically analyzed short-term morphological, physiological, and gene expression changes to elucidate the potential mechanisms underlying yield enhancement under different laser spectra. The results indicated that, compared to AL, all laser treatments (BL, CL, and DL) significantly increased root fresh weight, dry weight, and nitrogen content in seedlings. Furthermore, the final yield was significantly improved in all laser-treated groups, with the CL treatment exhibiting the highest yield. Transcriptome sequencing identified 10,497, 10,441, 10,700, and 10,757 expressed genes in the AL, BL, CL, and DL groups, respectively. Comparative analysis revealed 101, 1645, and 2247 differentially expressed genes (DEGs) in the BL/AL, CL/AL, and DL/AL comparisons, respectively. Gene Ontology (GO) enrichment analysis showed that these DEGs were significantly enriched in pathways such as metabolic processes, nitrogen metabolism, and protein amino acid phosphorylation. Notably, genes involved in the regulation of nitrogen compound metabolism were significantly upregulated in the CL and DL treatments. Further analysis of nitrogen metabolism and photosynthesis pathways revealed that laser irradiation induced the upregulation of specific genes. Interestingly, although physiological assays showed no significant changes in CAT, SOD, and POD activities, the expression of their corresponding genes was upregulated by laser treatment, suggesting these genes play a regulatory role during the supplemental lighting process. Therefore, our results indicated that laser supplemental lighting during the rice seedling stage increased the nitrogen content in plants and modulated the expression of related genes, and these changes might have been associated with the subsequent increase in rice yield. This study lays a foundation for understanding the molecular mechanisms of laser supplemental lighting and provides empirical support for the application of laser technology as an effective light source in agriculture.

## 1. Introduction

Plant supplemental lighting technologies have been increasingly applied in agricultural production. Laser technology, characterized by its monochromaticity, coherence, and high energy density, represents a distinct approach to delivering light stimuli in plant science research [1,2]. While conventional artificial light sources—particularly light-emitting diodes (LEDs)—are widely adopted for their efficiency, uniformity, and cost-effectiveness in large-scale applications, they typically emit broader wavelength bands [3,4]. Consequently, lasers offer complementary advantages due to their exceptional spectral purity, making them ideal for investigating fundamental plant responses to specific narrow-band light [5,6].

The synergistic effects of red and blue light components are particularly critical for plant physiology [7]. Red light (650–670 nm) corresponds closely to the absorption peaks of chlorophyll, directly driving photosynthesis, while blue light (450–470 nm) regulates photomorphogenic responses through photoreceptors such as cryptochromes and phototropins [8,9]. In rice, modulating the red-to-blue ratio has been shown to influence key agronomic traits, including RuBisCO activation and the expression of genes involved in flavonoid synthesis [10,11]. At the molecular level, specific wavelengths can enhance the repair cycle of Photosystem II and affect the cyclic electron flow around Photosystem I, thereby influencing photosynthetic efficiency [12,13,14]. Concurrently, blue light mediates auxin distribution by regulating PIN protein localization, intricately linking light signaling with developmental patterning [15,16]. Although the phenotypic plasticity of plants in response to light quality is well-documented [17,18], the precise transcriptional mechanisms through which defined light signals, particularly from monochromatic sources like lasers, interface with the core regulatory networks in rice remain inadequately explored. This knowledge gap currently limits the rational design of precision light regimes for smart agriculture [19,20].

Lasers exhibit diverse regulatory effects across different plant species [5]. In soybeans, high-intensity blue light preferentially activates genes involved in nodule formation and biological nitrogen fixation, while concurrently suppressing the expression of flowering-related genes [21,22]. Conversely, red light promotes stem elongation through gibberellin-pathway genes but impairs nodule development [23,24]. Ultraviolet laser treatment has been shown to activate stress-resistance gene networks, thereby enhancing disease resistance in plants [1]. In tomato cultivation, dynamically optimized red–blue light combinations coordinately regulate photoreceptor and sink-strength–related genes, achieving a balance between light capture and fruit biomass accumulation [25,26]. Pulsed ultraviolet lasers specifically induce flavonoid biosynthetic genes, leading to targeted enhancement of fruit antioxidant capacity [27,28]. In lettuce, red-light–dominant conditions stimulate the expression of genes involved in biomass accumulation but simultaneously inhibit nitrate metabolism [29,30], whereas blue light supplementation activates nitrate reductase genes, thereby optimizing both growth performance and food safety [31,32]. Collectively, these studies demonstrate that laser-based light treatments can modulate plant growth and development by regulating gene expression. However, much of the current evidence comes from vegetables and other horticultural species. Due to constraints related to operational conditions, cultivation scale, and field management, research on supplemental laser lighting in major field crops remains relatively limited and requires further systematic investigation.

Although laser-based supplemental lighting has been shown to play an important role in regulating plant growth and development [5,33], it remains unclear whether different red-to-blue light ratios exert similar or distinct effects, and its impact on field crops is still largely unexplored. In this study, we used the LGI-660/450 dual-wavelength semiconductor laser system to conduct supplemental lighting experiments on the rice cultivar Jijing129 during the seedling stage, a critical phase for stand establishment. Three red–blue light ratio treatments were applied: BL (R:B = 50:50; 150:150 μmol·m^−2^·s^−1^), CL (R:B = 60:40; 180:120 μmol·m^−2^·s^−1^), and DL (R:B = 75:25; 225:75 μmol·m^−2^·s^−1^), with natural light (AL) serving as the control. By comparing the effects of laser supplementation and natural light on rice traits and yield, and by analyzing morphological, physiological, and gene expression changes in Jijing129 under different light ratios, this study provides a foundation for further in-depth research on the mechanisms of laser-based supplemental lighting.

## 2. Results

### 2.1. Effects of Short-Term Laser Irradiation on Root Morphology and Yield in Rice

**Figure 1:**
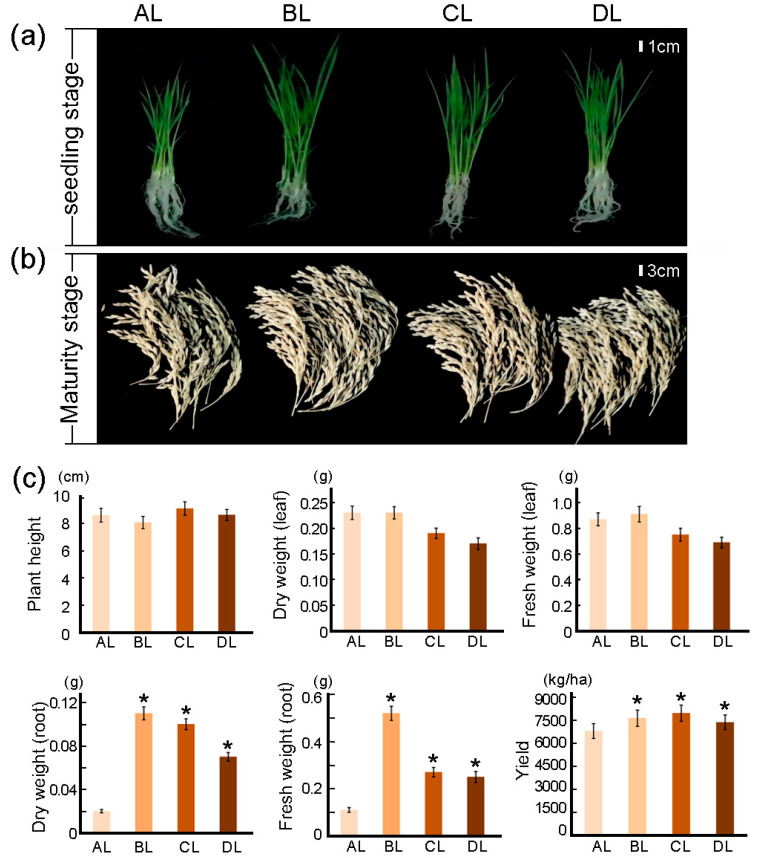
Results of rice phenotype and morphological index measurements under laser irradiation. (**a**) Jijing129 rice was irradiated with different light intensity ratios (AL, BL, CL, DL) for 28 days at both the seedling and maturity stages. (**b**) Root dry weight, root fresh weight, and actual yield of Jijing129 rice. (**c**) Morphological indices including plant height, leaf dry weight, leaf fresh weight, root dry weight, root fresh weight, and grain yield of Jijing129 rice under different treatments. The abbreviations BL, CL, and DL refer to the treatment groups BL (R50:B50; 150:150 μmol m^−2^ s^−1^), CL (R60:B40; 180:120 μmol m^−2^ s^−1^), and DL (R75:B25; 225:75 μmol m^−2^ s^−1^), while AL represents the control under natural light conditions. * indicates a statistically significant difference compared with the control AL group (* indicates *p* < 0.05).

We applied laser-based supplemental lighting to Jijing129 rice seedlings in a green house, using natural light as the control (AL). Three red-to-blue light ratios were tested: BL (RED: BLUE = 50:50; 150:150 μmol·m^−2^·s^−1^), CL (RED: BLUE = 60:40; 180:120 μmol·m^−2^·s^−1^), and DL (RED: BLUE = 75:25; 225:75 μmol·m^−2^·s^−1^). The results showed that, compared with AL, all laser treatments significantly increased root fresh weight and root dry weight (*p* < 0.05) (Figure 1a), and grain yield was also significantly improved (*p* < 0.05) (Figure 1b,c). Among the three laser regimes, DL led to the smallest improvements in both root biomass and yield relative to BL and CL. The significant increase in root dry weight across all laser treatments (*p* < 0.05) indicates that laser supplementation promotes dry matter accumulation in roots, possibly through enhanced dry-matter accumulation in root tissues.

Regarding yield, all treatment groups showed significant increases (Figure 1b), with the CL treatment exhibiting the most notable effect. This suggests that CL has a strong optimizing influence on rice yield formation than BL and DL (Table S1). The BL and DL groups also demonstrated significantly higher yields than the AL control, further confirming that laser irradiation positively influences yield development. In contrast, above-ground traits such as plant height, leaf fresh weight, and leaf dry weight showed no significant differences among treatments (Figure 1c). These results demonstrate that the application of laser supplemental lighting during the seedling phase significantly influences both root biomass and final yield in Jijing129.

### 2.2. Effect of Short-Term Laser Irradiation on the Small Organic Compounds Content in Rice

**Figure 2:**
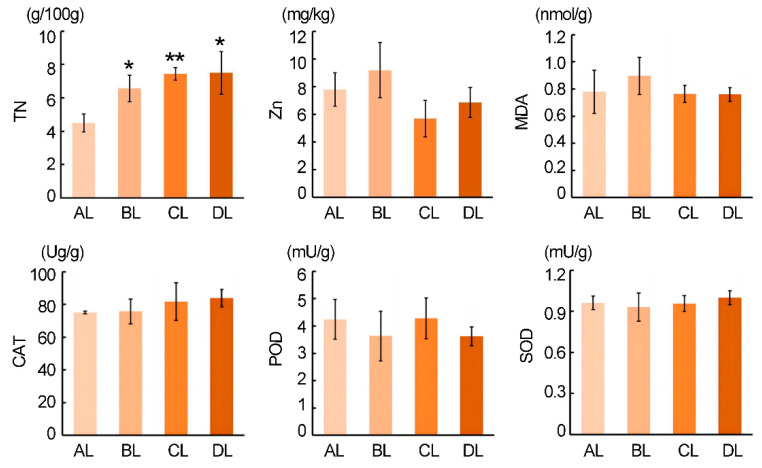
The variation in physical characteristics induced by laser irradiation. The abbreviations BL, CL, and DL refer to the treatment groups BL (R50:B50; 150:150 μmol m^−2^ s^−1^), CL (R60:B40; 180:120 μmol m^−2^ s^−1^), and DL (R75:B25; 225:75 μmol m^−2^ s^−1^), while AL represents the control under natural light conditions. *, ** indicates *p* < 0.05 and *p* < 0.01, respectively.

To investigate the alterations in small organic compounds content in rice plants subjected to short-term laser irradiation, we measured the contents of total nitrogen, zinc, and trace organic compounds in both the roots and leaves of Jiling129 rice under different treatments. The results (Figure 2) showed that, across treatments (AL, BL, CL, DL), the total nitrogen (TN) content gradually increased. Notably, the TN levels in the BL, CL, and DL groups were significantly higher than in the AL control (*p* < 0.05), suggesting that different laser treatments may promote nitrogen accumulation in the plants.

We also measured the contents of zinc and malondialdehyde (MDA), as well as the activities of catalase (CAT), superoxide dismutase (SOD), and peroxidase (POD). Although the measured values varied among treatments, no statistically significant differences were detected among the AL, BL, CL, and DL groups. These physiological indicators showed no obvious changes, which indirectly indicates that the primary effect of laser supplemental lighting during the seedling stage is to significantly enhance nitrogen content in Jijing129 seedlings. Meanwhile, although Figure 2 suggests a trend of increasing TN from BL to CL to DL, statistical comparisons among the laser treatments did not reveal significant differences (*p* > 0.05), indicating that the nitrogen enhancement may be primarily attributable to laser irradiation per se rather than to the specific red–blue ratio.

### 2.3. Effects of Short-Term Laser Irradiation on Genome-Wide Gene Expression in Rice

**Figure 3:**
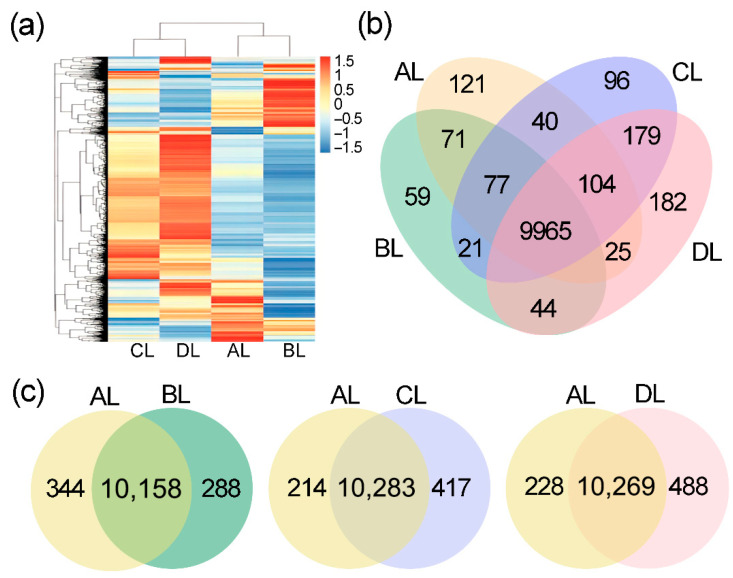
The variation in gene expression induced by laser irradiation. (**a**) Heatmap showing hierarchical clustering of gene expression profiles across AL, BL, CL, and DL samples. (**b**) Venn diagram displaying the number of co-expressed and specifically expressed genes among AL, BL, CL, and DL groups. (**c**) Pairwise Venn diagrams illustrating co-expressed genes between AL and each laser treatment group (BL, CL, and DL). The abbreviations BL, CL, and DL refer to the treatment groups BL (R50:B50; 150:150 μmol m^−2^ s^−1^), CL (R60:B40; 180:120 μmol m^−2^ s^−1^), and DL (R75:B25; 225:75 μmol m^−2^ s^−1^), while AL represents the control under natural light conditions.

In this study, we examined genome-wide gene expression changes in rice leaves after 28 days of laser irradiation using RNA-seq (Table S2). The results revealed (Figure 3a) that the AL, BL, CL, and DL samples expressed 10,401, 10,237, 10,480, and 10,497 genes, respectively. To identify common and unique gene expression patterns among the samples, Venn diagram analysis was conducted (Figure 3b). As shown, 9965 genes were co-expressed in all samples, while 121 genes were specifically expressed in AL, 59 genes in BL, 96 genes in CL, and 182 genes uniquely in AL, respectively. Furthermore, by analyzing the number of co-expressed and differentially expressed genes using Venn diagrams (Figure 3c), 10,283 genes were co-expressed in both the AL and CL groups. In comparison, 417 genes were specifically expressed in CL, and 214 genes were uniquely expressed in AL. The results observed genome-wide variations in both treatment-specific and treatment-specific gene expression responses following laser irradiation. These findings highlight the complexity of the transcriptional response to laser treatment across different rice genotypes.

### 2.4. Comparative Analysis of Differentially Expressed Genes in Rice Under Laser Irradiation

**Figure 4:**
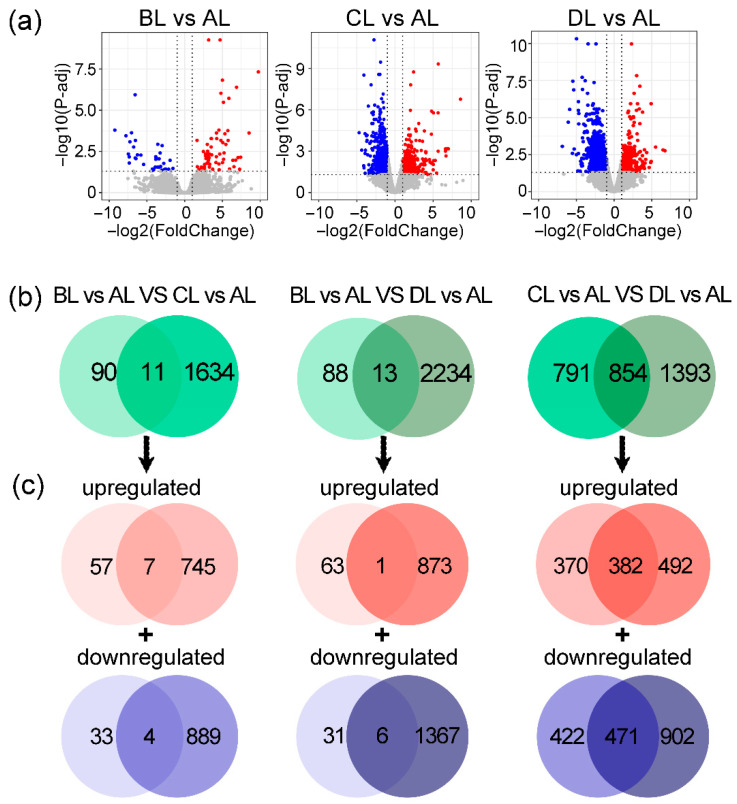
Changes in differentially expressed genes (DEGs) in the rice genome induced by different laser treatments. (**a**) Volcano plot displaying statistically significant up-regulated (red) and down-regulated (blue) DEGs. (**b**) Venn diagram illustrating overlapping and unique DEGs across three comparative groups (vs. control). (**c**) Shared and group-specific up-/down-regulated DEGs among the three comparisons. The abbreviations BL, CL, and DL refer to the treatment groups BL (R50:B50; 150:150 μmol m^−2^ s^−1^), CL (R60:B40; 180:120 μmol m^−2^ s^−1^), and DL (R75:B25; 225:75 μmol m^−2^ s^−1^), while AL represents the control under natural light conditions.

To further study the gene variation, we analyzed the differentially expressed genes (DEGs) under light conditions (BL, CL, and DL) and compared them with the control (AL). We quantified up- and down-regulated genes and characterized their functional enrichment. The results demonstrated that different light treatments significantly influenced gene expression patterns. Specifically, 101 DEGs were identified in the BL vs. AL comparison, consisting of 64 up-regulated and 37 down-regulated genes; 1645 DEGs were detected in CL vs. AL, with 752 up-regulated and 893 down-regulated genes; and 2247 DEGs were identified in DL vs. AL, including 874 up-regulated and 1373 down-regulated genes (Figure 4a).

Venn diagram analysis was used to explore the overlapping and specific DEGs among the groups (Figure 4b,c). Among the up-regulated genes, seven were commonly up-regulated in both BL vs. AL and CL vs. AL, with 57 specific to BL vs. AL and 745 specific to CL vs. AL. Additionally, one gene was up-regulated in both BL vs. AL and DL vs. AL, with 63 genes specific to BL vs. AL and 873 specific to DL vs. AL. For the comparison between CL vs. AL and DL vs. AL, 382 genes were commonly up-regulated, with 370 specific to CL vs. AL and 492 specific to DL vs. AL.

Regarding down-regulated genes, four were common between BL vs. AL and CL vs. AL, with 33 specific to BL vs. AL and 889 specific to CL vs. AL. Between BL vs. AL and DL vs. AL, 6 shared down-regulated genes were identified, along with 31 specific to CL vs. AL and 1367 specific to DL vs. AL. For the comparison of CL vs. AL and DL vs. AL, 471 DEGs were commonly down-regulated, with 422 specific to CL vs. AL and 902 specific to DL vs. AL.

Overall, the most pronounced gene down-regulation was observed under BL treatment, whereas CL and DL induced a greater number of up-regulated genes. These findings suggest that different light conditions exert distinct regulatory effects on gene expression.

### 2.5. Comparative Analysis of GO and KEGG Enrichment in Rice Under Laser Irradiation

**Figure 5:**
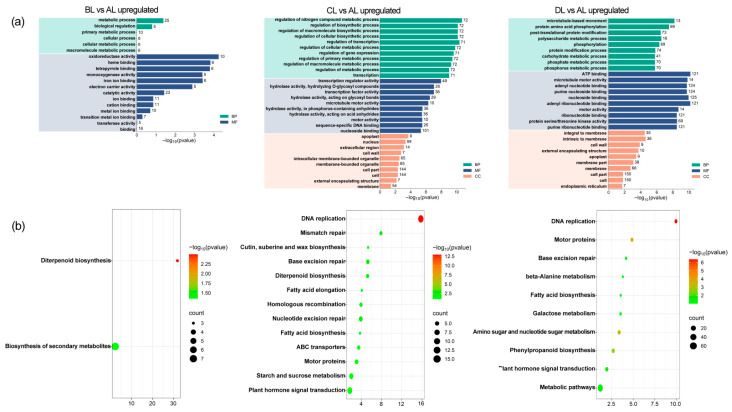
GO (**a**) and KEGG (**b**) enrichment analysis of rice up-regulated DEGs in response to laser irradiation. The abbreviations BL, CL, and DL refer to the treatment groups BL (R50:B50; 150:150 μmol m^−2^ s^−1^), CL (R60:B40; 180:120 μmol m^−2^ s^−1^), and DL (R75:B25; 225:75 μmol m^−2^ s^−1^), while AL represents the control under natural light conditions.

This study analyzed Gene Ontology (GO) enrichment in rice leaves subjected to various laser treatments. Analysis of up-regulated DEGs across different groups (Figure 5). In the comparison between AL and BL, the most enriched GO term in the biological process (BP) category was “metabolic process” (GO:0008152). In contrast, in the molecular function (MF) category, it was “oxidoreductase activity” (GO:0016491) (Figure 5a).

In the AL vs. CL comparison, the most enriched BP GO term was “regulation of nitrogen compound metabolic process” (GO:0051171), followed by “regulation of biosynthesis” (GO:0009889), “regulation of macromolecular biosynthesis” (GO:0010556), and “regulation of cell biosynthesis” (GO:0031326). The most significantly enriched cellular component (CC) GO terms were “apoplast” (GO:0048046) and “nucleus” (GO:0005634), while in the MF category, “transcriptional regulation activity” (GO:0030528) was predominant (Figure 5a).

For the AL vs. DL comparison, the most enriched BP GO terms were “microtubule-based movement” (GO:0007018) and “protein amino acid phosphorylation” (GO:0006468). The top CC GO terms included “components of the light membrane” (GO:0016021) and “membrane intrinsic” (GO:0031224), whereas in MF, “ATP binding” (GO:0005524) was most prominent. Additionally, highly enriched activities included “microtubule motor activity” (GO:0003777), “adenylate binding” (GO:0030554), and “purine nucleoside binding” (GO:0001883) (Figure 5a, Table S3).

To further elucidate the metabolic pathways involved, Kyoto Encyclopedia of Genes and Genomes (KEGG) enrichment analysis was conducted (Figure 5b, Table S4). In the AL vs. BL comparison, pathways related to diterpenoid biosynthesis and secondary metabolite biosynthesis were significantly enriched. In the AL vs. CL comparison, DNA replication and mismatch repair pathways were notably enriched. Meanwhile, in the AL vs. DL comparison, DNA replication and motor protein pathways demonstrated significant enrichment.

By integrating GO and KEGG analyses, we observed that up-regulated genes involved in nitrogen regulation were notably affected by laser irradiation. Specifically, the activity related to “regulation of nitrogen compound metabolic process” (GO:0051171) was significantly increased in the AL vs. CL comparison.

Down-regulated genes under BL condition exhibited distinct functional profiles. Similarly, we analyzed down-regulated genes in the differential expression groups, with results presented in Figure S1 (Tables S5 and S6) demonstrating significant enrichment of pathways, such as ‘photosynthesis’ (FDR < 0.05).

### 2.6. Laser Irradiation Differentially Regulates Nitrogen-Related Genes

**Figure 6:**
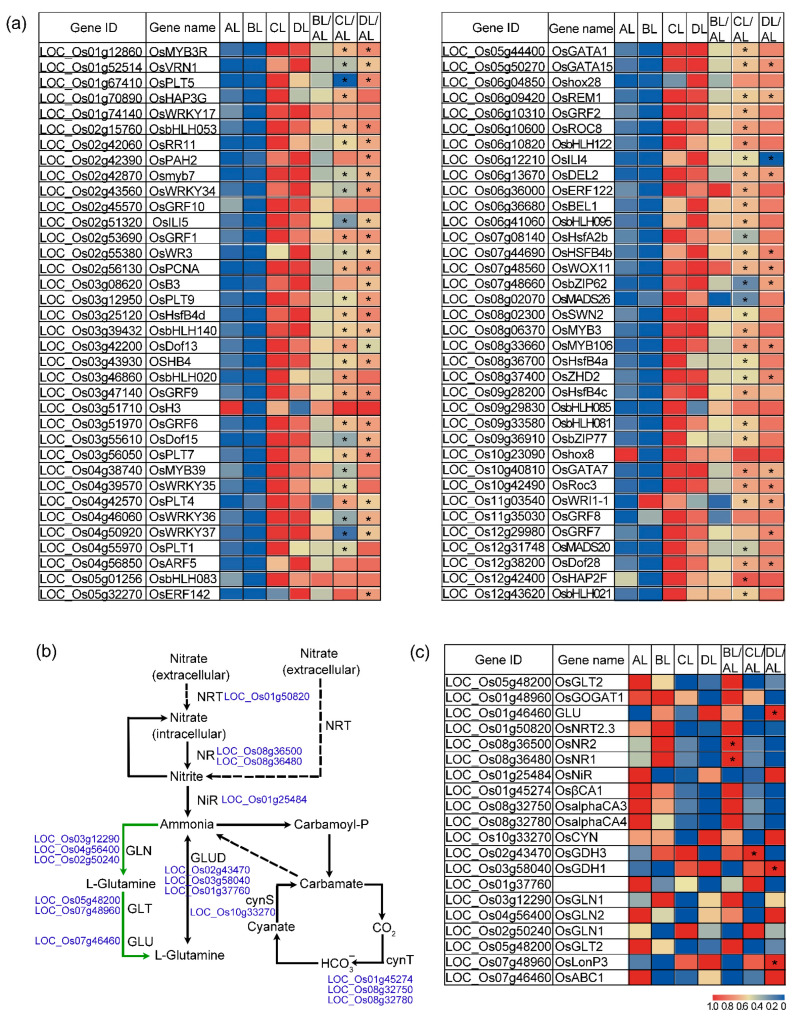
Differential response of nitrogen metabolism-related genes to short-term laser irradiation in rice. (**a**) Expression of genes involved in the regulation of the nitrogen compound metabolic process. (**b**) KEGG nitrogen metabolism pathway map. (**c**) Expression patterns of genes associated with the nitrogen metabolism pathway. The abbreviations BL, CL, and DL refer to the treatment groups BL (R50:B50; 150:150 μmol m^−2^ s^−1^), CL (R60:B40; 180:120 μmol m^−2^ s^−1^), and DL (R75:B25; 225:75 μmol m^−2^ s^−1^), while AL represents the control under natural light conditions. * indicates a statistically significant difference compared with the AL control group (* indicates *p* < 0.05).

GO enrichment analysis revealed that genes upregulated by laser irradiation were significantly enriched in the “regulation of nitrogen compound metabolic process” pathway (GO term). Expression levels and fold changes for all genes in this pathway were visualized using a heatmap (Figure 6a, Table S7). The results demonstrated a widespread and significant upregulation of these genes, particularly pronounced in the CL/AL and DL/AL comparison groups.

KEGG pathway enrichment analysis identified that genes downregulated by laser irradiation were significantly enriched in the “nitrogen metabolism” pathway (Figure S1, Figure 6b). Further analysis of the genes involved in this pathway revealed that down-regulated genes predominated between the three red–blue light ratios and the control. Interestingly, however, a subset of genes was significantly up-regulated: in particular, *OsNR1* and *OsNR2* were markedly up-regulated under the BL treatment; *OsGDH3* was significantly up-regulated under CL; and *OsGDH1* and *GLU* were significantly up-regulated under DL (Figure 6c).

These results indicate that the changes in nitrogen content in jijing129 seedlings following supplemental laser treatment are likely closely associated with a set of significantly differentially expressed genes, including both those directly involved in nitrogen metabolism and those regulating it. Furthermore, the roles and contributions of these genes may vary under different red–blue light ratios.

### 2.7. Laser Irradiation Differentially Regulates Photosynthesis-Related Gene Expression in Rice

**Figure 7:**
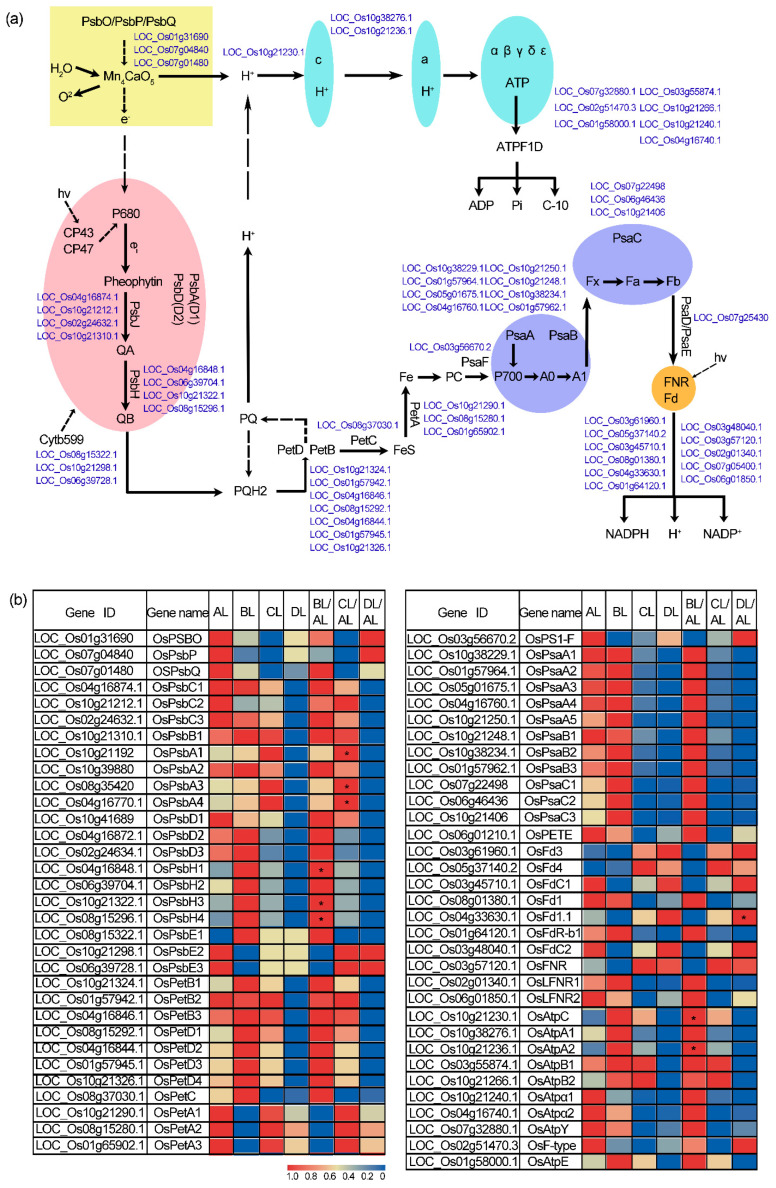
Differential regulation of rice photosynthetic pathway genes by laser irradiation. (**a**) KEGG photosynthesis pathway map. (**b**) The heatmap shows gene expression and fold change. Gene abbreviations: PSII (photosystem II), Pet (plastoquinone-electron transporter), PSI (photosystem I), ATPase (ATP synthase). The abbreviations BL, CL, and DL refer to the treatment groups BL (R50:B50; 150:150 μmol m^−2^ s^−1^), CL (R60:B40; 180:120 μmol m^−2^ s^−1^), and DL (R75:B25; 225:75 μmol m^−2^ s^−1^), while AL represents the control under natural light conditions. * indicates a statistically significant difference compared with the AL control group (* indicates *p* < 0.05).

Although we did not measure physiological parameters directly related to photosynthesis, GO and KEGG enrichment analyses revealed that photosynthesis-related pathways were significantly enriched (Figure S1). Consequently, we further analyzed genes within the photosynthesis pathway (Figure 7, Table S8). The results indicated that gene expression patterns exhibited distinct trends under different red–blue light ratios. Specifically, *OsPsbH1*, *OsPsbH3*, *OsPsbH4*, *OsAtpC*, and *OsAtpA2* were significantly upregulated under the BL treatment. In the CL treatment, *OsPsbA1*, *OsPsbA3*, and *OsPsbA4* were significantly upregulated, whereas *OsFd1.1* was significantly upregulated under the DL treatment.

PsbH is one of the major subunits of the Photosystem II (PSII) reaction center complex and is closely associated with the assembly, stability, and electron transport efficiency of PSII [34]. In contrast, AtpC and AtpA2 encode key subunits of chloroplast ATP synthase, which directly participate in ATP synthesis during the process of photophosphorylation [35]. The *PsbA* gene family encodes the D1 protein of the PSII reaction center, one of the core components responsible for primary charge separation and electron transfer [36]. *OsFd1.1* encodes ferredoxin, which is located at the acceptor side of Photosystem I (PSI) and serves as a key electron carrier linking the light reactions to multiple downstream metabolic pathways, such as carbon assimilation, nitrate reduction, and reactive oxygen species scavenging [37].

These results suggest that supplemental laser irradiation induces the expression of these genes during the rice seedling stage, thereby participating in the regulation of photosynthesis.

### 2.8. Laser Irradiation Differentially Regulates Rice Genes Associated with Physical Characteristics

**Figure 8:**
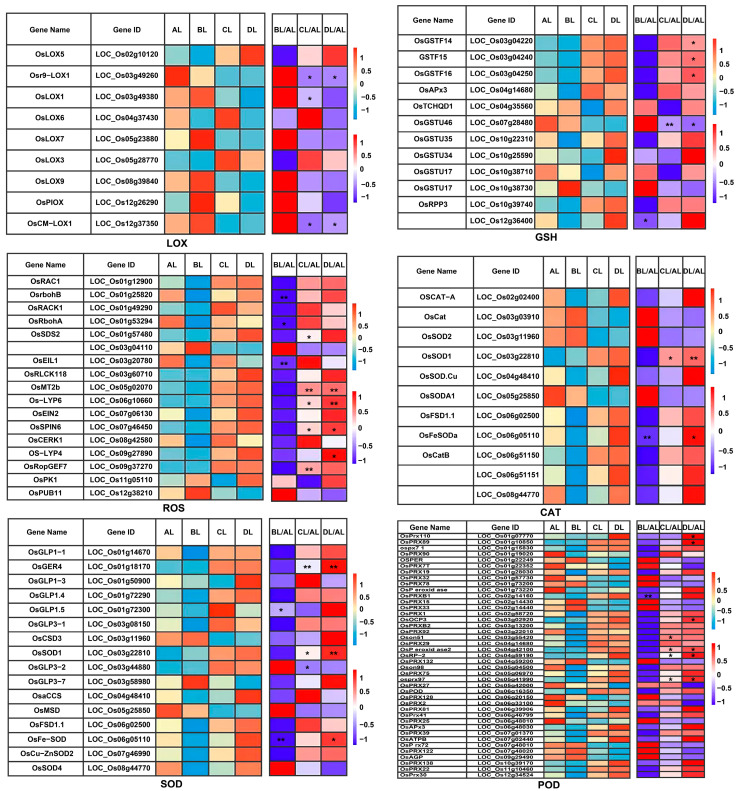
Heatmaps of gene expression related to antioxidant and signaling regulatory genes in rice under laser irradiation. The abbreviations BL, CL, and DL refer to the treatment groups BL (R50:B50; 150:150 μmol m^−2^ s^−1^), CL (R60:B40; 180:120 μmol m^−2^ s^−1^), and DL (R75:B25; 225:75 μmol m^−2^ s^−1^), while AL represents the control under natural light conditions. * and ** indicate statistically significant differences compared with the AL control group at *p* < 0.05 and *p* < 0.01, respectively.

Although no significant changes were detected in physiological indices such as CAT, SOD, and POD in this study, GO and KEGG enrichment analyses of the DEGs revealed that they were primarily enriched in pathways related to redox processes. Based on these findings, we further analyzed genes associated with lipoxygenase (LOX), glutathione (GSH), reactive oxygen species (ROS), and the activities of CAT, SOD, and POD (Figure 8, Table S9). The results indicated that under laser treatment, the expression of a subset of these genes was induced in rice seedlings, and their expression patterns differed significantly under different red–blue light ratios. Specifically, a considerable proportion of these genes were significantly upregulated or downregulated under laser treatment, suggesting that laser supplemental lighting can elicit molecular responses related to lipid peroxidation, the glutathione cycle, and ROS scavenging systems.

## 3. Discussion

In this study, we applied laser supplementary lighting with different red/blue ratios at the seedling stage of rice, and compared seedling morphology, physiological parameters, and gene expression under three light combinations (red: blue = 50:50, 60:40, and 75:25). Grain yield was further evaluated at maturity. The results showed that all three laser treatments increased root biomass in rice seedlings. The enhancement of root dry weight may indicate that laser exposure promoted dry matter accumulation in roots, a phenomenon that has also been reported in wheat and other crops under optimized light conditions [38]. Consistently, the anise study [39] demonstrated that He–Ne laser irradiation significantly increased the biomass of both anise sprouts and mature plants, confirming its growth-promoting effect, which is in line with our expectations.

However, that anise study also showed that different developmental stages (fruits, sprouts, and mature plants) displayed stage-specific responses to laser treatment in terms of pigments, total nutrients, minerals, vitamins, essential oils, and phenolic compounds. In contrast, in our seedling-focused measurements, among all physiological parameters examined, only nitrogen content was significantly increased, whereas other indicators such as SOD, POD, CAT, and MDA showed no obvious changes. In flax, the study [40] reported that combined treatment with laser and 6-benzylaminopurine (BAP) enhanced nitrogen metabolism and increased the activities of key nitrogen assimilation enzymes (GS, GOGAT, and GDH). In agreement with this, we also observed an increase in nitrogen content in our rice plants, together with up-regulation of the *GDH gene* under the CL and DL laser treatments.

The discrepancy between transcript levels and the corresponding enzyme activities may reflect the multilayered nature of metabolic regulation. On the one hand, changes in mRNA abundance can be buffered at the protein level and therefore may not markedly affect enzyme activity; on the other hand, many key metabolic enzymes are subject to post-translational regulation, such as phosphorylation, redox modification, or allosteric control, which can amplify or attenuate the effects of transcriptional changes.

Moreover, laser supplementation improved the grain yield of the rice cultivar Jijing129, which is consistent with the findings of a previous study [1]. That study showed that, under pot conditions in the field, low-intensity laser treatment increased both the number of effective tillers and grain yield per plant, with a 16.8% increase in effective panicle number and a 9.01% increase in per-plant yield. Similarly, in our field trial, overall yield was enhanced, with the most pronounced improvement observed under the CL treatment (red: blue = 60:40). Notably, a 60:40 red/blue ratio can improve photosynthetic efficiency and yield without markedly altering vegetative growth, a result that has already been validated in sweet pepper [38].

It is noteworthy that the transcriptomic responses exhibited a substantial disparity in the number of DEGs among treatments: only 101 DEGs were detected in BL, compared with 1645 and 2247 in CL and DL, respectively, despite the relatively modest phenotypic differences. This pattern suggests that the specific red–blue ratios in CL and DL may trigger much broader photoreceptor-mediated transcriptional reprogramming, whereas BL may represent a relatively stable or balanced state that requires only limited transcriptional adjustment. Although minor variation among biological replicates cannot be completely excluded and may have contributed to the absolute DEG counts to some extent, the pronounced quantitative contrast among treatments is unlikely to be explained solely by experimental variation. Instead, it most likely reflects genuinely different regulatory strategies adopted by plants under the distinct laser light conditions.

The DEGs identified under CL/AL treatment were significantly enriched in the pathway of “regulation of nitrogen metabolic process.” Further analysis revealed that laser irradiation under both CL and DL conditions markedly up-regulated the expression of a large number of genes, including several classes of transcription factors, such as members of the WRKY family (*OsWRKY17*, *OsWRKY34*, *OsWRKY35*, *OsWRKY36*, *OsWRKY37*), the bHLH family (*OsbHLH020*, *OsbHLH021*, *OsbHLH053*, *OsbHLH081*, *OsbHLH083*, *OsbHLH085*, *OsbHLH095*, *OsbHLH140*), and the MYB family (*OsMYB3*, *OsMYB3R*, *OsMYB7*, *OsMYB39*, *OsMYB106*). These transcription factors are likely to act together within transcriptional regulatory networks that govern stress responses, hormone signaling, and plant growth and development. The functions of some of these genes have been relatively well characterized. For example, *OsMYB3* is a key R2R3-MYB gene that controls anthocyanin biosynthesis and accumulation in the pericarp of black rice [41]. In contrast, many members of the WRKY, bHLH, and MYB families identified here have not yet been subjected to systematic genetic and mechanistic studies. Their pronounced up-regulation under both CL and DL treatments suggests that they may be involved in the regulation of nitrogen metabolism, responses to light conditions, and adaptation to environmental stresses. Therefore, future functional studies are warranted to elucidate their roles and to explore their potential utility in improving nitrogen use efficiency and enhancing stress tolerance in rice.

The analysis showed that, under different red–blue light ratios, photosynthesis-related genes exhibited markedly distinct expression patterns, displaying a typical “light-quality-specific response” profile. Specifically, under the blue-light-dominant BL treatment, the expression of *OsPsbH1*, *OsPsbH3*, *OsPsbH4*, *OsAtpC*, and *OsAtpA2* was significantly upregulated. PsbH proteins are important subunits of the photosystem II (PSII) reaction center complex and are closely associated with PSII assembly, stability, and electron transport efficiency [34]. AtpC and AtpA2 encode key subunits of chloroplast ATP synthase and are directly involved in ATP production during photophosphorylation [35]. The coordinated upregulation of these genes suggests that, under BL conditions, plants may simultaneously enhance PSII reaction center function and energy conversion efficiency, thereby improving the capacity of rice seedlings to utilize light energy.

Under the CL treatment, *OsPsbA1*, *OsPsbA3*, and *OsPsbA4* were markedly upregulated. The *PsbA* gene family encodes the D1 protein of the PSII reaction center, which is a core component responsible for primary charge separation and electron transfer, and is also the protein most prone to damage and frequent turnover under photoinhibitory conditions [36]. The strong induction of multiple *PsbA* copies under CL conditions may be associated with accelerated D1 turnover, enhanced PSII repair capacity, and increased tolerance to photoinhibition, thereby helping to maintain stable operation of the photosynthetic apparatus under higher light input or specific light-quality environments.

In contrast, under the DL treatment with a higher proportion of red light, *OsFd1.1* was significantly upregulated. *OsFd1.1* encodes ferredoxin, located at the terminal acceptor side of PSI, which serves as a key electron carrier linking the light reactions to multiple downstream metabolic pathways, such as carbon assimilation, nitrate reduction, and reactive oxygen species scavenging [37]. Its induced expression under DL conditions may help increase the flow of electrons from PSI into various metabolic routes, optimize the distribution of reducing power within the cell, and thereby coordinate the balance between photosynthetic electron transport and metabolic processes.

Taken together, the differential regulation of genes encoding PSII structural proteins, ATP synthase subunits, and ferredoxin under distinct red–blue light ratios may represent an important molecular basis underlying the effects of laser light supplementation on photosynthetic performance and growth of rice seedlings. Under laser supplemental lighting during the seedling stage of jijing129, an in-depth elucidation of the regulatory mechanisms of nitrogen metabolism pathways and key genes such as *PsbA* and *OsGDH3* should be a primary focus of future research.

Although no significant changes in the activities of antioxidant enzymes such as CAT, SOD, and POD were detected at the physiological level in this study, GO and KEGG enrichment analyses of the transcriptome, together with the expression patterns of related genes, indicated that rice seedlings had already mounted a clear molecular response in redox and antioxidant pathways under laser supplemental lighting.

On the one hand, this phenomenon of “transcriptional responses preceding detectable physiological changes” may reflect a preventive regulatory strategy, whereby plants grown under different red–blue laser light ratios fine-tune the expression of genes associated with LOX, GSH metabolism, ROS scavenging, and antioxidant enzymes (CAT, SOD, POD) to mitigate potential oxidative stress in advance. Such regulation likely keeps ROS levels and the extent of lipid peroxidation within a range that is insufficient to cause marked changes in enzyme activities. On the other hand, the pronounced divergence in expression patterns of these genes under different red–blue light ratios suggests that laser light quality not only affects photosynthetic electron transport and energy metabolism but may also reshape the dynamic balance between ROS production and scavenging, thereby establishing light-quality-specific antioxidant regulatory schemes.

Taken together, although conventional physiological indices did not show significant differences, the transcriptomic data reveal an underlying molecular regulatory network activated by the combined effects of laser illumination and light quality, providing the clues for further elucidating how red–blue light ratios modulate cellular redox homeostasis and defense responses.

## 4. Materials and Methods

### 4.1. Plant Materials and Treatments

Jijing129 rice (*Oryza sativa L*. ssp. japonica) was used in this study. Seeds were surface-sterilized with 75% ethanol for 30 s followed by 2% sodium hypochlorite for 10 min, then germinated on moist filter paper at 28 °C in darkness for 3 days. Uniformly germinated seeds were transferred to a greenhouse for seedling cultivation. During the seedling stage, the temperature in the greenhouse was controlled at 28±2 °C during the day and 20±2 °C at night, with the relative humidity maintained at 60–70%. Laser supplemental lighting with different red-to-blue light ratios was applied during the seedling period. When the seedlings reached an appropriate height for transplanting, they were transplanted to experimental plots (total area 0.5 ha, with separate plots for each treatment) located in Zhenlai Township, Baicheng City, Jilin Province, where they were grown under conventional field management practices until maturity for yield determination.

The laser treatment system (Model LGI-660/450, Changchun Optical Instrument Co., Changchun, China) consisted of diode lasers emitting at 660 nm (red) and 450 nm (blue), with total photon flux density maintained at 300 μmol m^−2^ s^−1^ at plant canopy level. The supplemental laser light treatments were defined by both the red: blue (R:B) ratio and the corresponding photon flux densities as follows: R50:B50 (150:150 μmol m^−2^ s^−1^), R60:B40 (180:120 μmol m^−2^ s^−1^), and R75:B25 (225:75 μmol m^−2^ s^−1^), with natural daylight (AL) serving as the control.

Both the laser-supplemented treatment and the control were cultivated in the same greenhouse under identical baseline environmental conditions (temperature, humidity, and natural photoperiod). The only difference between them was that the control received no laser supplemental lighting, whereas the treatment group received additional laser light on top of the natural daylight. The irradiation schedule (1:00–9:00 and 17:00–23:00) was designed to provide supplemental lighting during periods of natural light deficiency while avoiding overlap with peak sunlight hours, ensuring that the treatment groups received a consistent, specific light source at fixed intervals.

Samples were collected at the three-leaf stage of rice seedlings (about 28 days after sowing), and the harvested tissues included leaves and roots. A portion of the samples was immediately flash-frozen in liquid nitrogen after collection and stored at −80 °C for subsequent RNA extraction and gene expression analyses.

Measurements of seedling plant height and morphological traits of leaves and roots were conducted with 10 biological replicates per treatment. The mean values of each parameter were calculated, and differences among treatments were statistically analyzed. Physiological parameters were measured using 5 biological replicates, with three technical replicates per sample; the mean of the technical replicates was used for statistical analysis of treatment differences. RNA-seq was performed with three biological replicates. qRT-PCR was conducted with three biological replicates, each with three technical replicates.

At the end of the growth cycle, rice plants from each treatment plot were harvested and yield was determined. For yield determination, harvested rice grains were manually weighed, and dry weight yield data (after moisture removal) were recorded and standardized to ensure that the results accurately reflected actual field productivity.

### 4.2. Determination of Small Organic Compounds, Enzyme Activity, and SOD and POD Activity

All biochemical assays were performed with five biological replicates and three technical replicates. Fresh leaf tissue (500 mg) was homogenized in respective extraction buffers for each assay.

LOX activity was measured by spectrometry according to the method of Zhang et al. [42]. 0.5 g of fresh sample was homogenized in ice-cold 50 mM sodium phosphate buffer (pH 6.5), centrifuged (12,000× *g*, 15 min, 4 °C). The supernatant was incubated with linoleic acid as the substrate, and the absorbance change at 234 nm was measured. GSH content was determined according to the method of Griffith [43]. The tissue was homogenized in 5% perchloric acid, centrifuged, and the supernatant was reacted with 5,5′-dithiobis (2-nitrobenzoic acid) (DTNB). The absorbance was measured at 412 nm, and the reduced glutathione (GSH) content was calculated. ROS level was assessed by measuring H_2_O_2_ content according to the method of Velikova et al. [44]. Antioxidant enzyme activities were measured as follows: Superoxide dismutase (SOD) activity at 560 nm based on nitroblue tetrazolium photoreduction inhibition [45]; Peroxidase (POD) activity at 470 nm using guaiacol-H_2_O_2_ system [46]; Catalase (CAT) activity at 240 nm by monitoring H_2_O_2_ decomposition [47]. All spectrophotometric measurements were conducted using a SmartSpec™ Plus spectrophotometer (Bio-Rad, Hercules, CA, USA).

### 4.3. RNA Extraction and Sequencing

Total RNA was extracted from the collected Jijing 129 rice tissue samples using a plant total RNA extraction kit (Qiagen RNeasy Kit, Suzhou, China). To ensure the reliability of subsequent experiments, we used previously reported RNA extraction and cDNA library construction methods and analyzed RNA quality by agarose gel electrophoresis [48]. Three biological replicates were used for each sample. The RNA samples that passed quality control were sequenced on a high-throughput sequencing platform (Illumina HiSeq 2000, San Diego, CA, USA). After sequencing, we removed the adapter sequence, poly(A) tail, and low-quality reads, yielding more than 4 GB of clean data with a 150 bp read length per sample. The Q20 and Q30 values for the sequencing data were both greater than 92%, indicating high data quality and suitability for subsequent analysis (Table S2). To further analyze the sequencing data, we used HISAT2 (version 2.2.1) to align the quality-controlled reads to the reference genome and performed statistical analysis of the alignment results using RSeQC (version 5.0.1) [49]. It establishes a sequence index containing more than 90% of the introns of known genes for short-fragment splicing reads and uses hierarchical indexing and multiple alignment strategies to address alignment issues for these reads. It has high alignment accuracy and fast running speed.

### 4.4. Identification and Enrichment Analysis of Differentially Expressed Genes

In this study, we systematically conducted selection and enrichment analyses of DEGs. The R package DESeq2 (version 1.42.0) was used for differential expression analysis. The criteria for selecting differentially expressed genes (DEGs) were MAX normalized counts > 30 (one of which was sufficient), |log2(FoldChange)| > 1, and *p*-value < 0.05 (Table S10). In terms of enrichment analysis, we determined the main biological functions of differentially expressed genes through GO functional significance enrichment analysis, in which the selection condition for significantly enriched GO terms was the corrected FDR value < 0.05. Similarly, the hypergeometric test was applied to each pathway in the Kyoto Encyclopedia of Genes and Genomes for enrichment analysis, and a corrected FDR < 0.05 was used as the threshold to identify significantly enriched metabolic pathways among the differentially expressed genes. These analyses helped us determine the biological functions of differentially expressed genes and the metabolic pathways they mainly affected, providing a basis for subsequent functional studies. In addition, we used the microbial bioinformatics platform (https://www.bioinformatics.com.cn/, accessed on 2 May 2025), TBTools (version 2.056), and RStudio (version 2024.04.0) to generate heat maps from experimental data for visualization. GO enrichment was analyzed using the online agriGOv2 platform (http://systemsbiology.cau.edu.cn/agriGOv2/index.php, accessed on 10 May 2025), and KEGG graph drawing was performed on the KEGG catalog website (http://www.genome.ad.jp/kegg/kegg2.html, accessed on 10 May 2025).

### 4.5. qRT-PCR Validation

To verify the reliability of RNA sequencing data, six DEGs were randomly selected for qRT-PCR analysis. The total RNA extraction method was as described above, and about 2 μg of total RNA was used to synthesize first-strand cDNA using gene-specific primers. The primer design was based on our previous study and obtained from the qPCR primer database (https://biodb.swu.edu.cn/qprimerdb/, accessed on 12 May 2025). The total volume of the qRT-PCR reaction system was 20 μL, using the Top Green qPCR SuperMix kit (Beijing Quanshijin Biotechnology Co., Ltd., Beijing, China), which included 10 μL of 2× TransStart^®^ Top Green qPCR SuperMix. Each sample was set up with three technical replicates and three biological replicates, and expression levels were normalized to the reference gene GADPH. The qRT-PCR results are shown in Figure S2. The primers are listed in Table S10. The results showed that qRT-PCR data were highly consistent with transcriptomic data, demonstrating the reliability of the latter.

### 4.6. Statistical Analysis

In this study, statistical data analysis and heat maps were generated based on correlation values or gene expression levels. Other physiological data were analyzed using one-way analysis of variance (ANOVA). Data are presented as the mean ± SE (standard error) of three, five or ten biological replicates. Data visualization was performed in GraphPad Prism version 9.0 (GraphPad Software, San Diego, CA, USA) or Microsoft Office Excel, with * *p* < 0.05 and ** *p* < 0.01 as significant.

## 5. Conclusions

This study investigated Jijing 129 rice by setting up three laser supplemental lighting treatments with different red-to-blue light ratios against a natural light control. It systematically compared the effects on morphological and physiological indicators, gene expression during the seedling stage, and yield at maturity, aiming to explore the potential mechanisms for yield enhancement.

The results demonstrated that, compared to natural light, the laser supplemental lighting treatments significantly promoted root growth, increased plant nitrogen content in seedlings, and ultimately led to an effective increase in yield at maturity. Notably, among all treatments, the CL (red-to-blue ratio 60:40) treatment exhibited the most significant yield-enhancing effect, indicating that different red-to-blue light ratios have a significant impact on the yield of jijing129 rice.

To further elucidate the underlying molecular mechanisms, we performed a differential gene expression analysis. The results revealed that laser treatment induced expression changes in several key genes involved in nitrogen metabolism, photosynthesis, and redox pathways (e.g., *OsNR1* and *OsNR2*). Importantly, the gene expression profiles induced by different red-to-blue light ratios were markedly different, suggesting that they may regulate rice growth and development through distinct molecular pathways.

The obtained data, together with the identified candidate genes and key pathways, provide an important theoretical basis and practical reference for further elucidating the molecular mechanisms of laser supplemental lighting and for carrying out precise light-environment regulation and variety improvement.

## Data Availability

The datasets generated and analyzed in this study are available at PRJNA1297612 (http://www.ncbi.nlm.nih.gov/bioproject/1297612, accessed on 28 July 2025).

## Supplementary Materials

The following supporting information can be downloaded at: https://www.mdpi.com/article/10.3390/plants14243712/s1, Figure S1: GO (a) and KEGG (b) enrichment analysis of rice down-regulated DEGs in response to laser irradiation. The abbreviations BL, CL, and DL refer to the treatment groups BL (R50:B50; 150:150 μmol m^−2^ s^−1^), CL (R60:B40; 180:120 μmol m^−2^ s^−1^), and DL (R75:B25; 225:75 μmol m^−2^ s^−1^), while AL represents the control under natural light conditions; Figure S2: qRT-PCR validation of transcriptome reliability. Bars: qPCR data; Lines: Transcriptome data. The abbreviations BL, CL, and DL refer to the treatment groups BL (R50:B50; 150:150 μmol m^−2^ s^−1^), CL (R60:B40; 180:120 μmol m^−2^ s^−1^), and DL (R75:B25; 225:75 μmol m^−2^ s^−1^), while AL represents the control under natural light conditions. Table S1: Yield assessment of rice cultivar Jijing129 under laser supplemental lighting with different red:blue ratios. Three red-to-blue light ratios were tested: BL (RED: BLUE = 50:50; 150:150 μmol·m^−2^·s^−1^), CL (RED: BLUE = 60:40; 180:120 μmol·m^−2^·s^−1^), and DL (RED: BLUE = 75:25; 225:75 μmol·m^−2^·s^−1^). Data are means ± SE (n ≥ 4). Means with different letters are significantly different (*p* < 0.05) within the same treatments. Asterisks (*) indicate significant differences between control and treatments of the same genotype which were determined by Student’s *t*-test (* *p* < 0.05); Table S2:The overview of RNA-seq data; Table S3: Upregulated results of GO analysis; Table S4: Upregulated results of KEGG analysis; Table S5: Downregulated results of GO analysis; Table S6: Downregulated results of KEGG analysis; Table S7: Expression analysis of nitrogen metabolism-related genes based on GO and KEGG; Table S8: Expression analysis of photosynthesis-related genes based on GO and KEGG; Table S9: Expression analysis of related genes LOX, GSH, ROS, CAT, SOD and POD; Table S10: DEGs data between different comparison groups; Table S11:List of primers used for qRT-PCR.
