# Supplementary material for: Transcriptomic Analysis of Rice (Jijing129) Reveals Growth and Gene Expression Responses to Different Red-Blue Laser Light Treatments
Source: Plants (Basel). 2025 Dec 5;14(24):3712. doi: 10.3390/plants14243712 (PMC12736977; doi:10.3390/plants14243712)
Supplement: Supplementary file 1 [file plants-14-03712-s001.zip › Table S1.pdf]

Table S1 Yield assessment of rice cultivar Jijing129 under laser supplemental lighting with different red:blue ratios. Three red-to-blue light ratios were tested: BL (RED: BLUE = 50:50; 150:150 $\mu mol \cdot m^{-2} \cdot s^{-1}$), CL (RED: BLUE = 60:40; 180:120 $\mu mol \cdot m^{-2} \cdot s^{-1}$), and DL (RED: BLUE = 75:25; 225:75 $\mu mol \cdot m^{-2} \cdot s^{-1}$). Data are means ±SE ($n \geqslant 4$). Means with different letters are significantly different ($p < 0.05$) within the same treatments. Asterisks (*) indicate significant differences between control and treatments of the same genotype which were determined by Student's t-test (*$p < 0.05$)

	control	Laser		
	AL	BL	CL	DL
Yield	13578.3+214.71	15244.2+241.04[*a]	15897+251.36[*b]	14709.9+232.60[*a]
