# Supplementary figures and images for: Transcriptomic Analysis of Rice (Jijing129) Reveals Growth and Gene Expression Responses to Different Red-Blue Laser Light Treatments
Source: Plants (Basel). 2025 Dec 5;14(24):3712. doi: 10.3390/plants14243712 (PMC12736977; doi:10.3390/plants14243712)

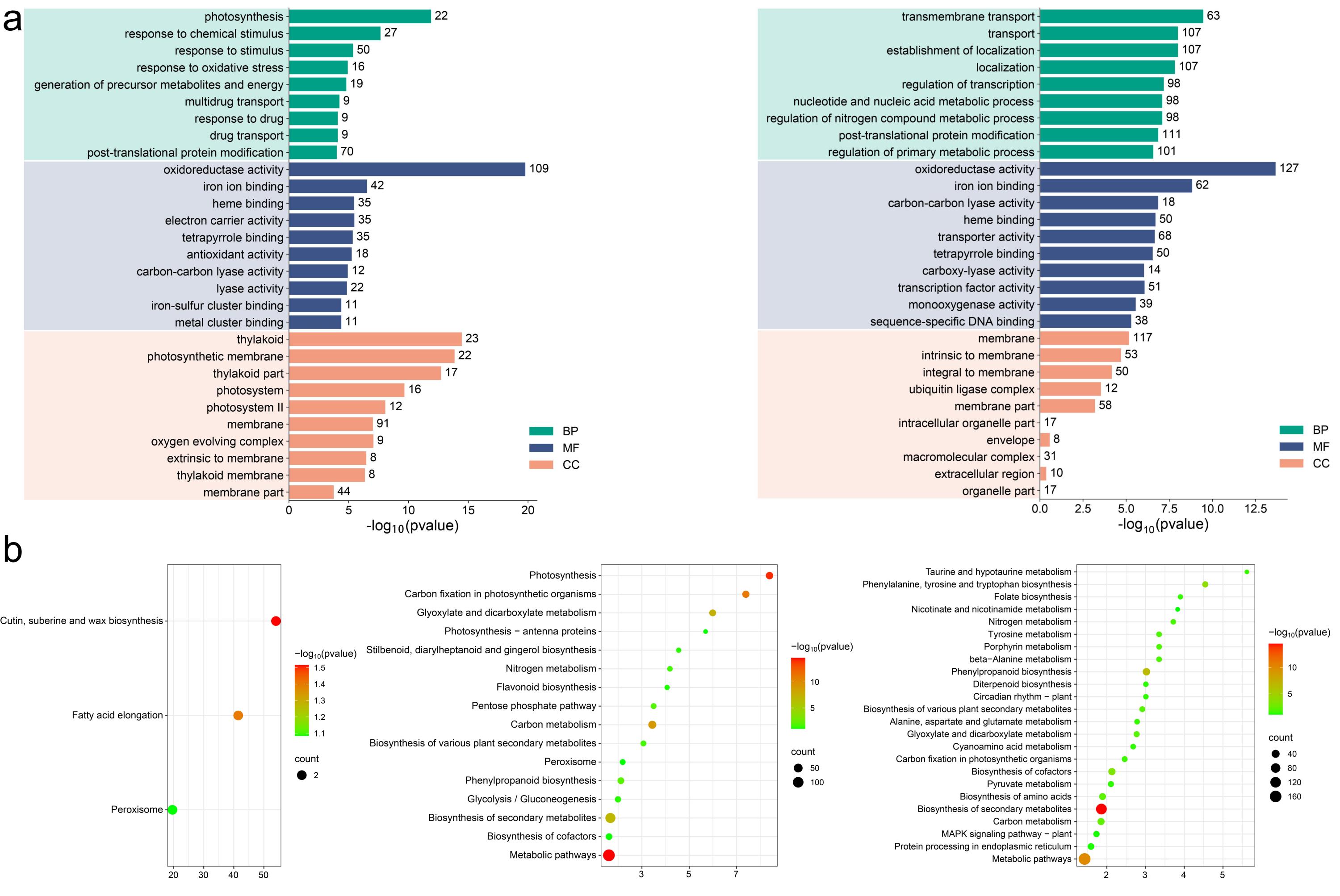

Supplement: Supplementary file 1 [file plants-14-03712-s001.zip › Figure S1.jpg]

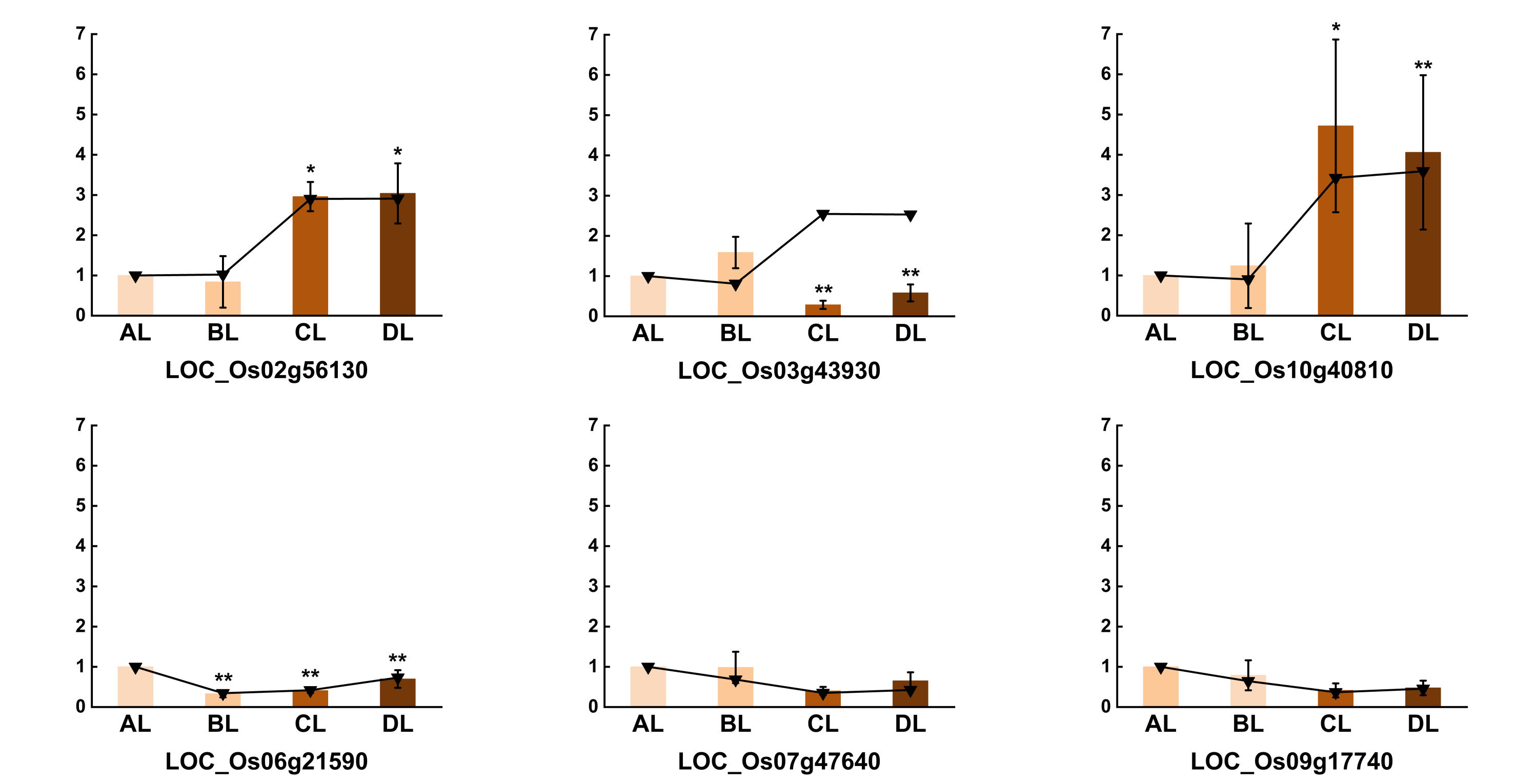

Supplement: Supplementary file 1 [file plants-14-03712-s001.zip › Figure S2.jpg]
